# Supplementary material for: The endosomal Q-SNARE, Syntaxin 7, defines a rapidly replenishing synaptic vesicle recycling pool in hippocampal neurons
Source: Commun Biol. 2021 Aug 18;4:981. doi: 10.1038/s42003-021-02512-4 (PMC8373932; doi:10.1038/s42003-021-02512-4)
Supplement: Supplementary file 2 — Supplementary Information [file 42003_2021_2512_MOESM2_ESM.pdf]

Supplementary Information

The endosomal Q-SNARE, Syntaxin 7, defines a rapidly replenishing synaptic vesicle recycling pool in hippocampal neurons

Yasunori Mori[1,#,*], Koh-ichiro Takenaka[1], Yugo Fukazawa[2] and Shigeo Takamori[1,#]

[1] Laboratory of Neural Membrane Biology, Graduate School of Brain Science, Doshisha University, Kyoto, Japan

[2] Division of Brain Structure and Function, Faculty of Medical Science, University of Fukui, Fukui, Japan

[#] All correspondence should be addressed to Y.M. (yasmori@yamanashi.ac.jp) or S.T. (stakamor@mail.doshisha.ac.jp)

[*] Present address: Department of Biochemistry, Faculty of Medicine, University of Yamanashi, Yamanashi, Japan

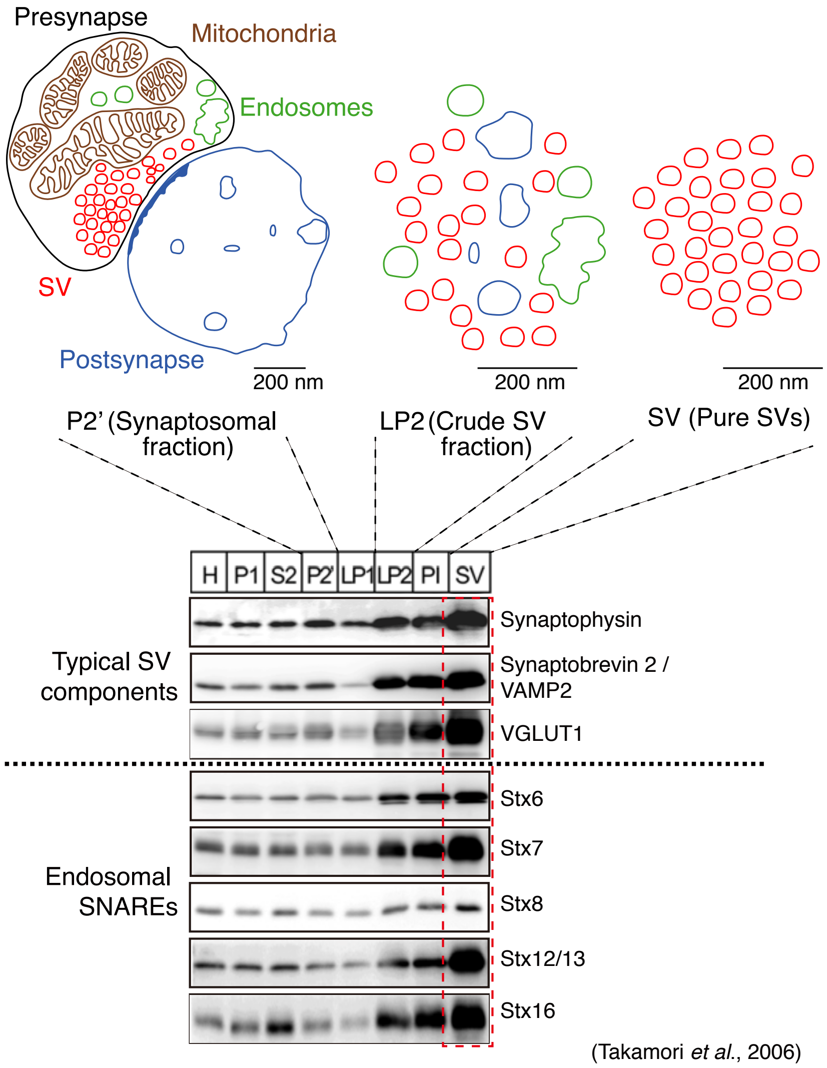

Supplementary Fig 1 Various endosomal SNAREs are enriched in the purified SV fraction from rat brains. Distributions of endosomal SNAREs analyzed by western blot in various fractions during SV purification (see Takamori et al., 2006). Note that endosomal SNAREs (Stx6, 7, 8, 12/13, 16) are co-enriched in the SV fraction with authentic SV proteins, such as Synaptophysin, Synaptobrevin 2/VAMP2, and vesicular glutamate transporter 1 (VGLUT1).

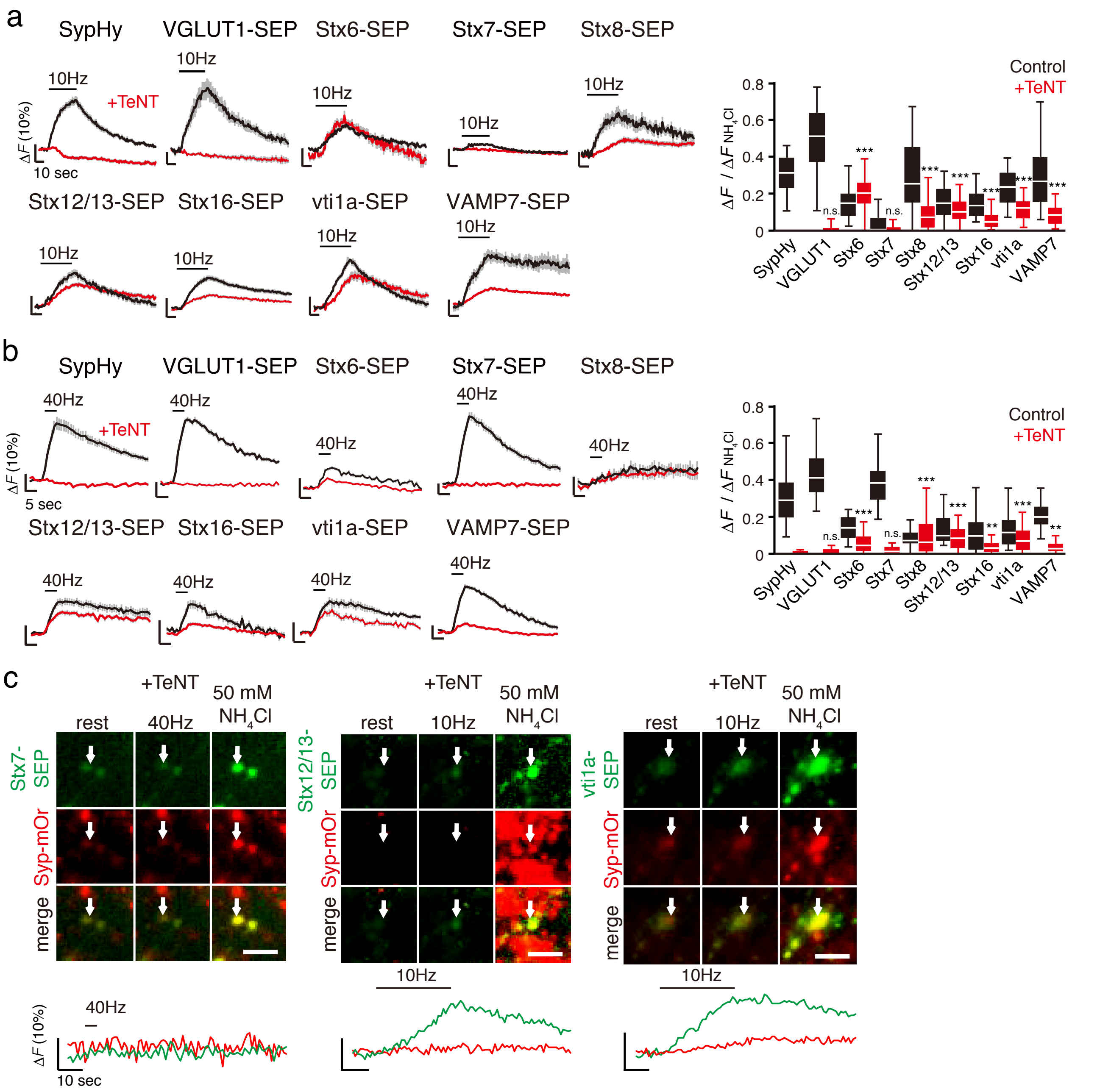

Supplementary Fig 2 Exocytic responses of endosomal SNARE-SEPs in cultured hippocampal neurons. Average traces of endosomal SNARE-SEP responses elicited by stimulation either at 10-Hz (**a**) or 40-Hz stimulation (**b**) with (red) or without (black) TeNT pretreatment are shown. SypHy and VGLUT1-SEP were used as controls for SV-resident proteins. Fluorescence was normalized to the values upon application of NH_4Cl. Right box-whisker plots show responses of SEPs without (black) and with (red) TeNT pretreatment. Data were obtained from >35-100 boutons for each condition. The boxes, the white lines in the boxes and the whiskers in these plots and hereafter indicate the first and third quartiles, the medians, and the minimum and maximum values, respectively. Percent inhibition of the SEP responses by TeNT in comparison to SypHy at 200APs was statistically analyzed. n.s indicates not significant ($p > 0.05$), ** $p < 0.01$, *** $p < 0.001$ compared to SypHy after TeNT treatment (Student's *t*-test). **c** Double imaging of SEP-fusion proteins (green) and Syp-mOr (red) after TeNT treatment. In all cases, Syp-mOr did not respond to stimulus after TeNT treatment, whereas Stx12/13-SEP (middle) and vti1a-SEP (right) showed significant responses by 10-Hz stimulation. Stx7-SEP (left) did not show detectable responses to 40-Hz stimulation after TeNT treatment. Traces represent fluorescence changes from the boutons indicated by arrows. Scale bars indicate 5 μm.

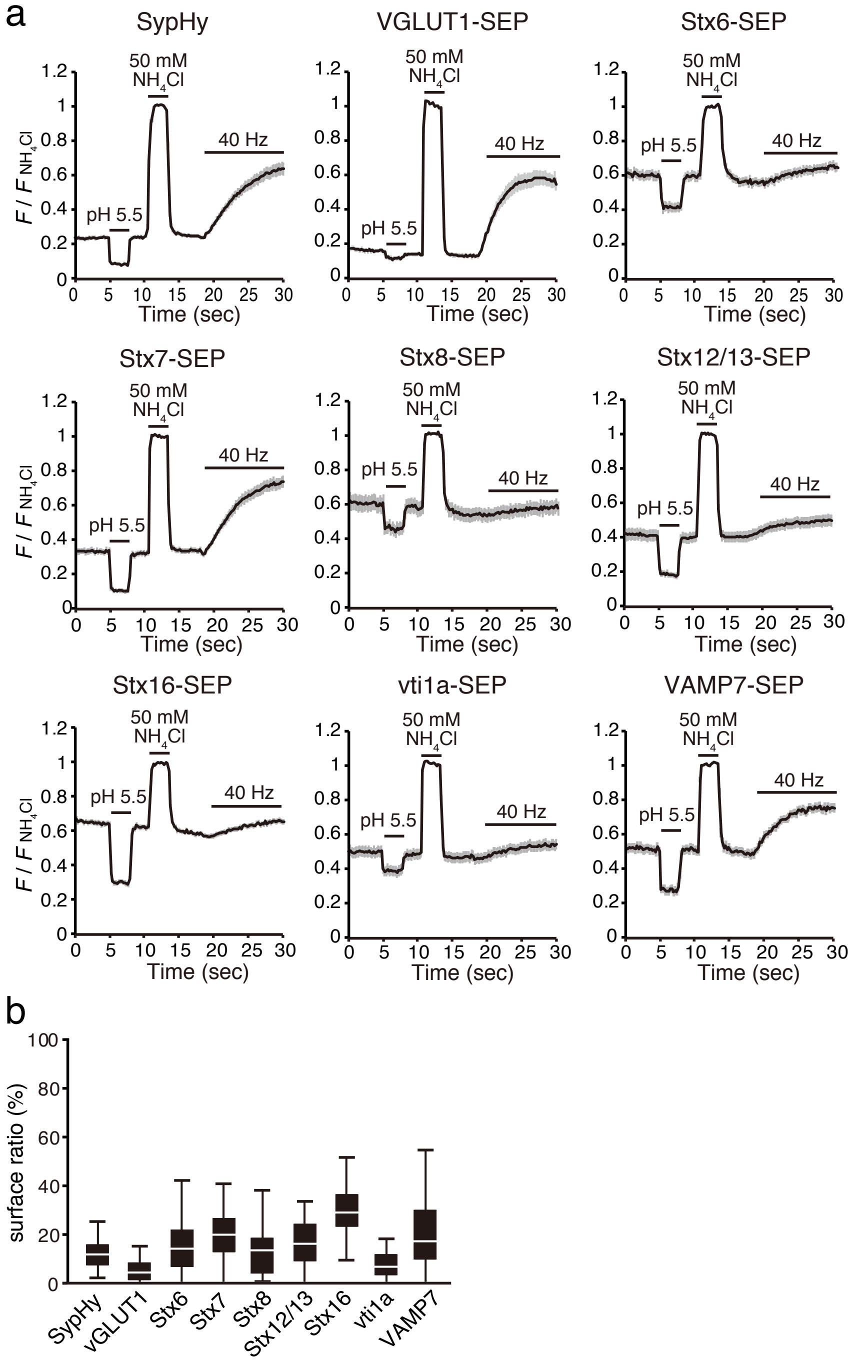

Supplementary Fig 3 Average traces of SEP-reporters to estimate surface fraction and vesicular pHs. a Average traces for estimation of the surface fraction of SEP probes and vesicular pH. To restrict the analysis exclusively to active synapses, 40-Hz stimulation (400 APs) was applied after subsequent application of an acidic solution (pH 5.5) and an NH_4Cl solution (pH 7.4). Values from traces were used to quantify vesicular pHs shown in Fig. 1h. **b** Surface fraction of SEP probes calculated from experiments shown in (**a**). Box-whisker plots show the surface fraction relative to total SEP probes.

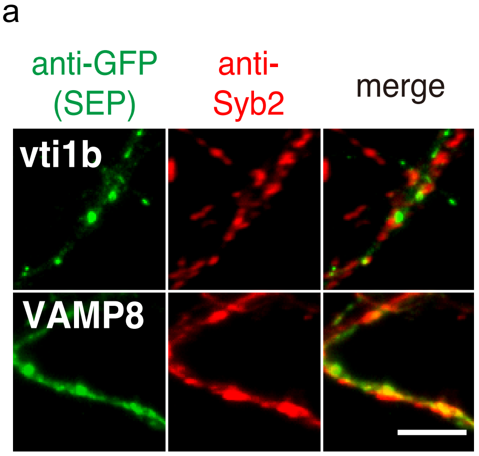

b

	responding puncta / total puncta	rate (%)
SypHy	516 / 545	94.7
Stx7-SEP	189 / 244	77.5
vti1a-SEP	115 / 348	33.0
VAMP8-SEP	24 / 188	11.3
vti1b-SEP	1 / 134	0.7

Supplementary Fig 4 Vti1b and VAMP8 do not localize to active synapses. a Representative images of vti1b-SEP and VAMP8-SEP (green) co-stained with Syb2 (red). Note that vti1b-SEP and VAMP8-SEP rarely colocalized with Syb2-positive puncta. Scale bar indicates 5 µm. **b** Quantification of responding puncta of SypHy, Stx7-SEP, vti1a-SEP, VAMP8-SEP and vti1b-SEP at 200APs at 40-Hz stimulation. All data were obtained from at least 10 images, and responding puncta were defined as stimulation-mediated fluorescence increases beyond 5% of the total SEP fluorescence revealed by NH_4Cl application.

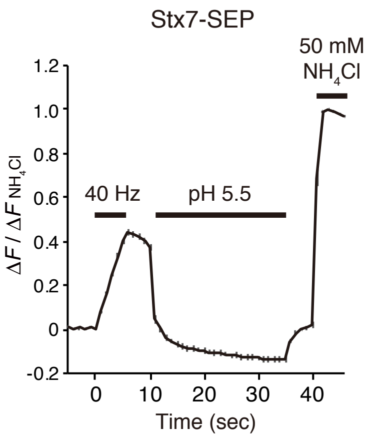

Supplementary Fig 5 Evidence for exocytosis of Stx7-SEP vesicles upon stimulation. An acidic solution (pH 5.5) was applied after 40-Hz stimulation (200 APs). Fluorescence was normalized by that during 50 mM NH_4Cl application at the end of recordings. Note that application of the acidic solution right after the cessation of stimulation largely quenched the fluorescence Stx7-SEP.

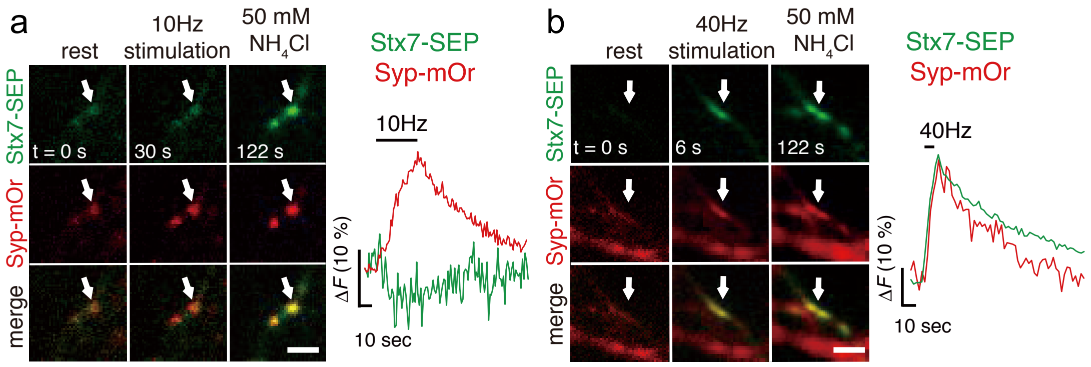

Supplementary Fig 6 Dual-color imaging of Stx7-SEP and Syp-mOr. Stx7-SEP and Syp-mOr were lentivirally transduced in the same culture preparations, and 10-Hz (**a**) and 40-Hz (**b**) stimulation (200 APs) were applied. Fluorescent images were taken before stimulation (rest, t = 0 s), at the end of stimulation (10-Hz stimulation, t = 30 s for **a**; 40-Hz stimulation, t = 6 s for **b**), and at the end of recordings (50 mM NH_4Cl, t =122 s for **a**; t = 122 s for **b**). Traces represent fluorescence changes from boutons indicated by arrows. Scale bars indicate 2.5 μm.

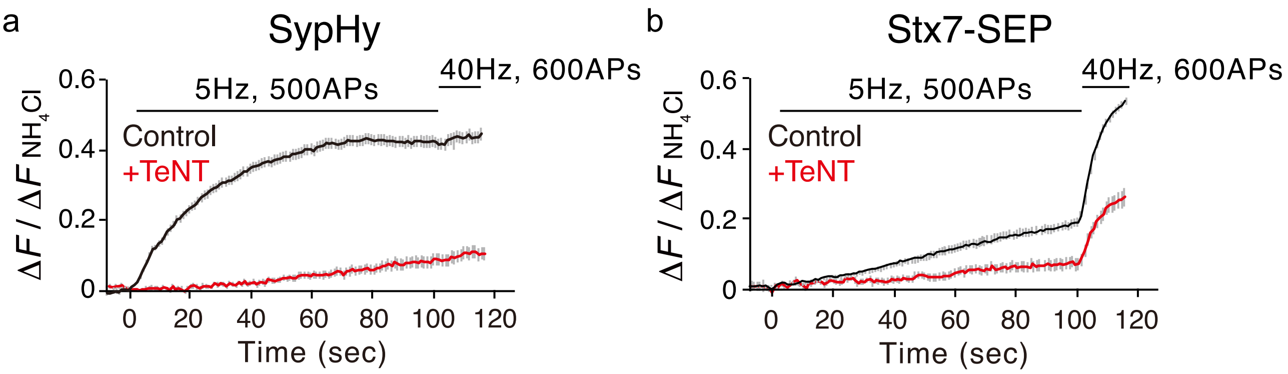

Supplementary Fig 7 TeNT-insensitive response of Stx7-SEP after prolonged pre-stimulation. Neurons expressing SypHy (**a**) or Stx7-SEP (**b**) were pretreated with 2 μM bafilomycin A, and were pre-stimulated with 500 APs at 5 Hz, and additionally stimulated with 600 APs at 40 Hz. Fluorescence was normalized by that during 50 mM NH_4Cl application. Traces of TeNT-pretreated neurons are shown in red and those of control neurons are shown in black. Data were obtained from >50-100 boutons for each condition.

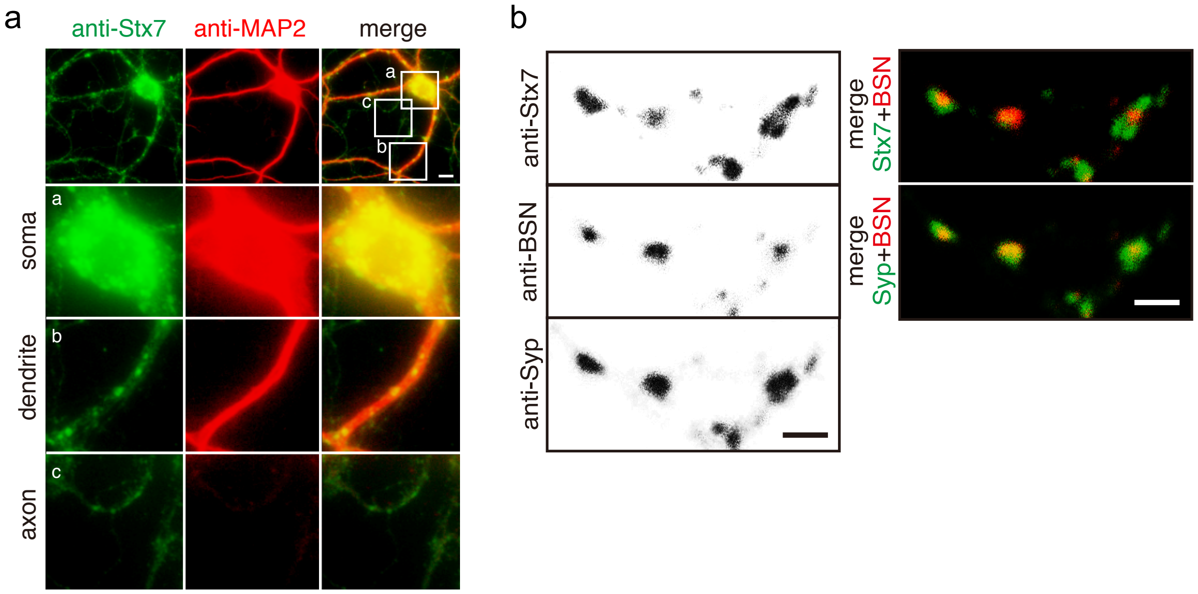

Supplementary Fig 8 Cellular distribution of endogenous Stx7 in cultured hippocampal neurons. a Neurons were double-immunostained for Stx7 (left panels, green) and a dendritic marker, MAP2 (middle, red). Panels in the second row (a), third row (b), and bottom row (c) are magnified views of the boxed areas in the upper right panel, representing soma, dendrite, and axon, respectively. Note that Stx7 immunoreactivities were apparent in the cell soma, dendrites (MAP2-positive neurites) and axon (MAP2-negative neurite). Scale bar indicates 5 μm. **b** Neurons were triple-immunostained for Stx7, Syp and an active zone marker, Bassoon (BSN) (same as Fig. 3b). Note that Stx7 immunoreactivities rarely overlapped with BSN signals. Scale bars indicate 1 μm.

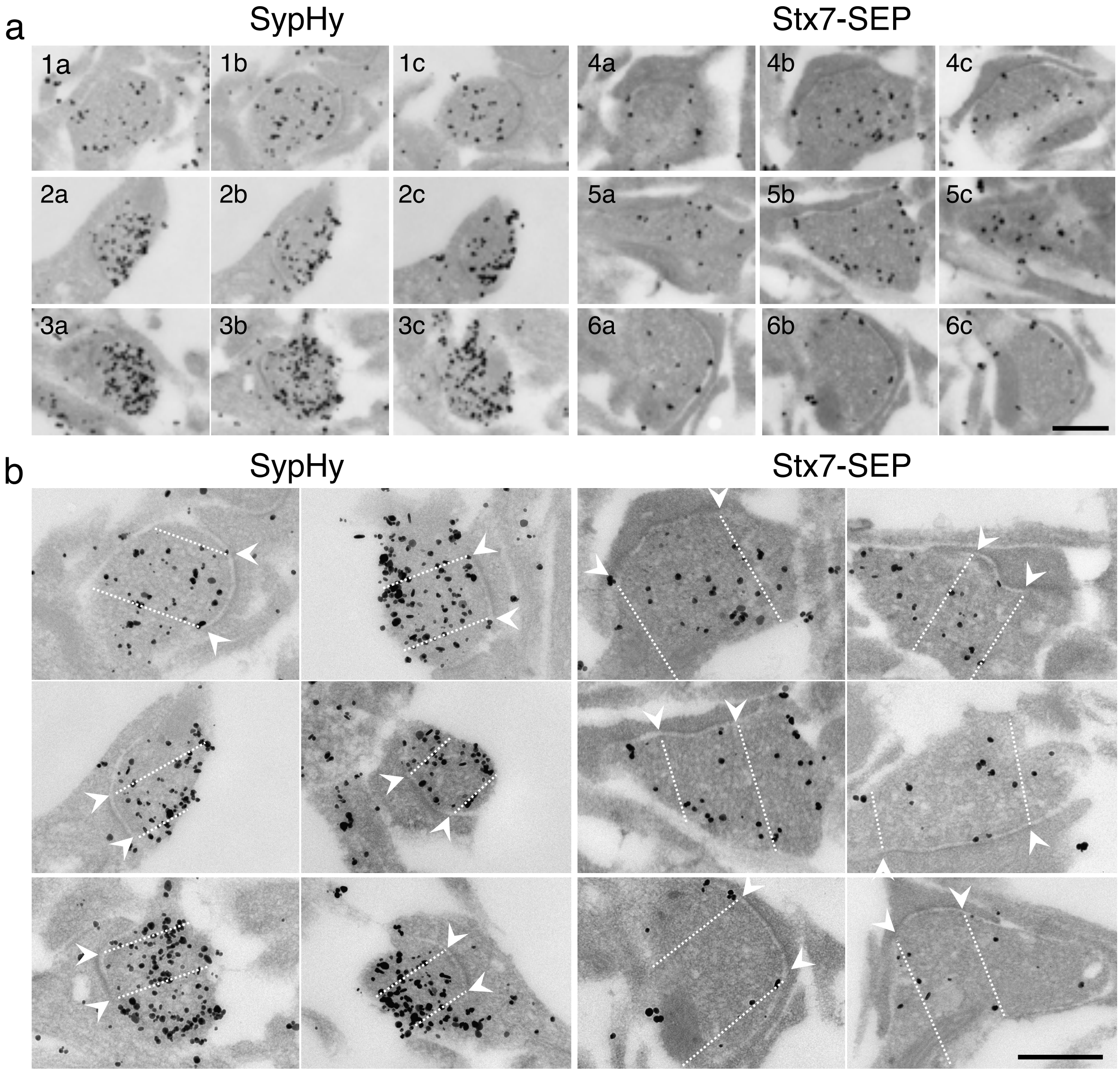

Supplementary Fig 9 Serial section images and analysis of immunoreactivity for SypHy and Stx7-SEP of cultured hippocampal neurons. Three consecutive electron micrograms of 3 representative synapses for SypHy and Stx7-SEP expressing neurons (**a**), and 6 example images used for the measurement of a distance to the nearest AZ for individual immunoparticles for each neuron are shown (**b**). Note that only a subset of neuronal profiles contains immunoparticles, and these are concentrated in presynaptic varicosities. Although PSD appears in several consecutive images in series images, only one image from each series shows clear synaptic cleft, and such images taken from the perpendicular direction to the synaptic cleft were selected for the measurement of the distance. The selected images were further processed by drawing two lines (white dashed lines) extended from both edges of AZs, which are deduced from the PSD structures. Immunoparticles enclosed by the borderlines were subjected for the distance measurement. White arrowheads indicate the edges of PSDs. Immunoparticles whose centers were located outside of the neuronal profile were considered as those originated from SypHy and Stx7-SEP expressed at the surface of the plasma membrane, and thus were excluded from the measurement. Scale bars in (a) and (b) indicate 300 nm.

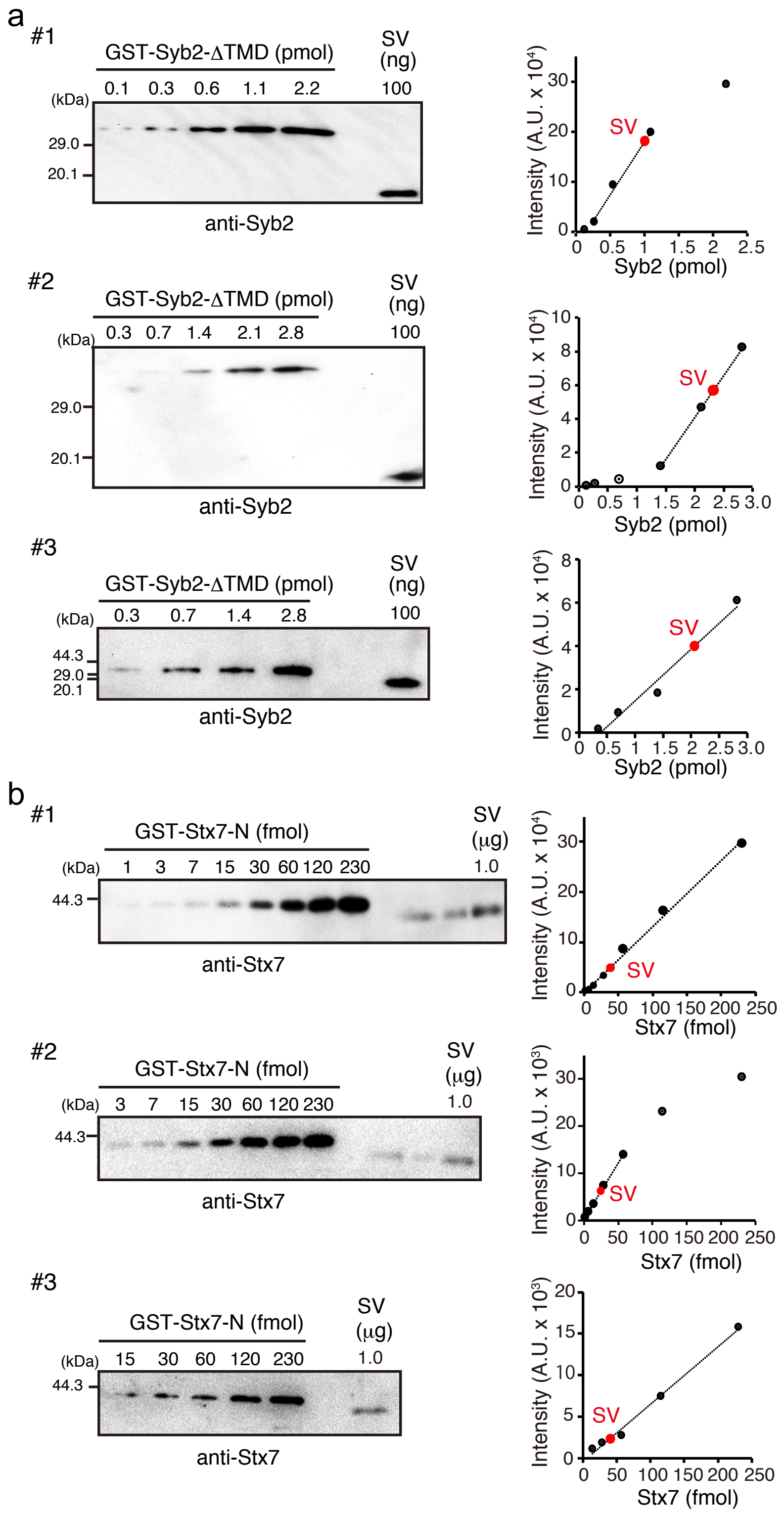

Supplementary Fig 10 Estimation of Stx7 copy number per SV. Quantitative western blot analysis for Syb2 (**a**) and Stx7 (**b**) are shown. Reference proteins (GST-Syb2-ΔTMD or GST-Stx7-N) and the pure SV fraction were subjected to western blotting using the respective specific antibodies. Band intensities were quantified with Quantity One ver. 4.6.9 software (Bio-Rad) and references within the linear ranges were used for standard curves. The vesicle concentration and protein concentration of the SV fraction used in measurements were 26.7 nM and 99.7 ng/μL, respectively. To justify the method, we estimated the copy number of Syb2 per SV to be 67.89 ± 23.51 (SD) which was highly consistent with previous studies[1,2].

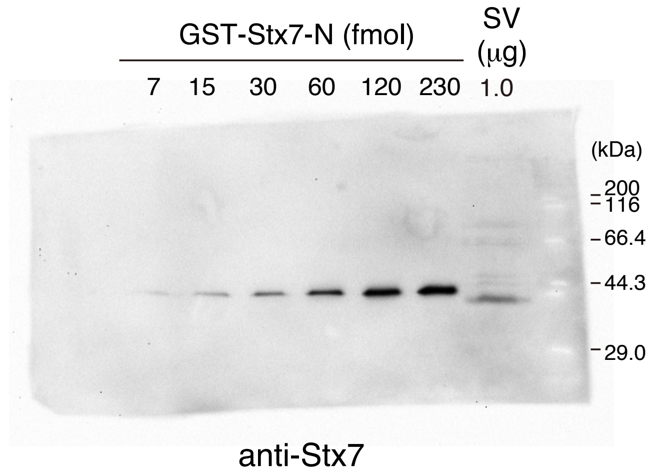

Supplementary Fig 11 Uncropped and unedited blot corresponding to Fig. 3g.

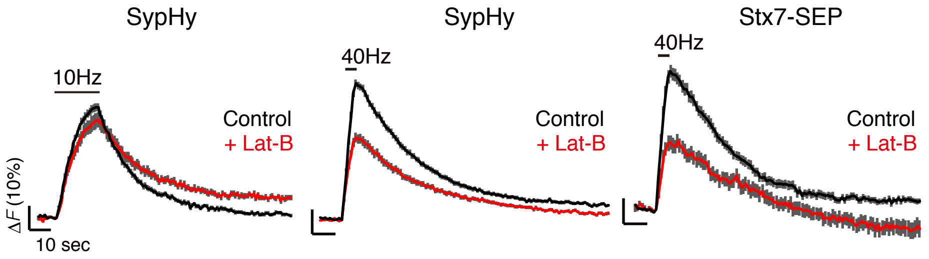

Supplementary Fig 12 Effect of latrunculin B on SypHy and Stx7-SEP responses. Neurons transfected with either SypHy (left and middle) or Stx7-SEP (right) were pretreated with 10 μM latrunculin B (Lat-B, red traces) for 1 min before being used for imaging experiments. Essentially the same results with Lat-A were obtained in which Lat-B caused decreased responses of SypHy and Stx7-SEP at 40 Hz, but did not affect the SypHy response at 10 Hz.

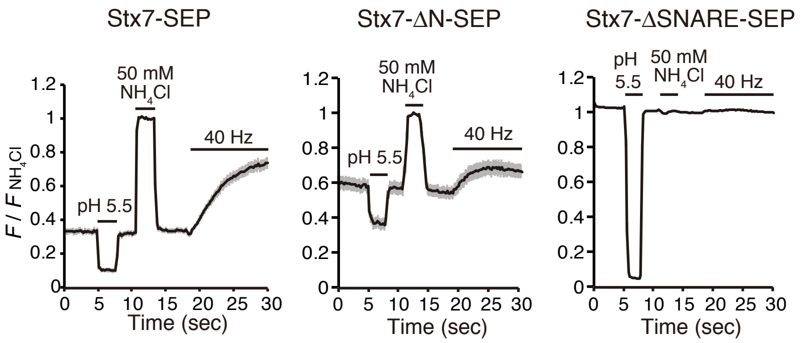

Supplementary Fig 13 Estimation of surface fraction and vesicular pH of Stx7 full-length and truncated mutants. Average fluorescence trace of Stx7-SEP (left), Stx7-ΔN-SEP (middle) and Stx7-ΔSNARE-SEP (right) in response to a sequential application of a pH 5.5 solution and a 50 mM NH_4Cl solution (pH 7.4). The fluorescence was normalized by those during 50 mM NH_4Cl application. Note that Stx7-ΔSNARE-SEP did not respond to stimuli since it exclusively localized to the cell surface.

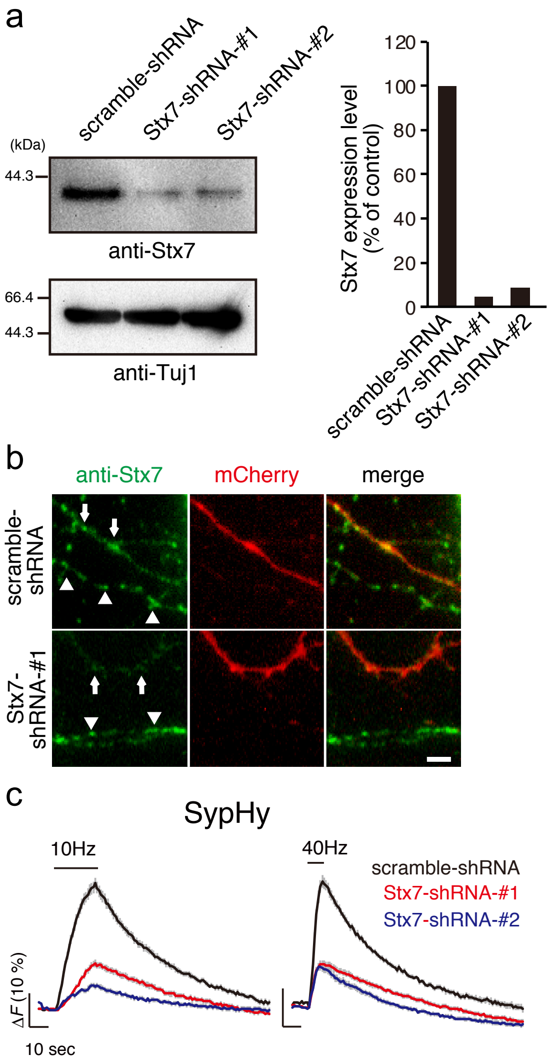

Supplementary Fig 14 Chronic knockdown of Stx7 results in retardation of SV recycling at both 10-Hz and 40-Hz stimulation. a Specific reduction of Stx7 expression by shRNA. Immunoblot analysis of lysates derived from hippocampal cultured neurons transfected with either control shRNA (scramble-shRNA) or with two independent shRNA for Stx7 (Stx7-shRNA#1 and #2). The upper blot shows an immunoblot with anti-Stx7 antibody, and the bottom blot shows an immunoblot with anti-Tuj1 antibody performed as a loading control. Each lane contains 2.5 μg protein. The right graph shows Stx7 expression level relative to control (scramble-shRNA). Signal intensities of Stx7 were normalized with respect to signal intensities of Tuj1. Note that Stx7 protein was effectively reduced by two shRNAs. **b** Specific reduction of Stx7 expression by shRNA. mCherry was co-expressed with shRNAs, so that neurons transduced with shRNA were identified by mCherry fluorescence. Arrows indicate neurites originating from a transduced neuron, whereas arrowheads indicate neurons originating from a non-transduced neuron. In control experiments (scramble-shRNA), Stx7-immunoreactivities were observed both in mCherry-positive and in mCherry-negative axons (upper panels). On the other hand, Stx7-immunoreactivities were substantially reduced in mCherry-positive axons compared to mCherry-negative axons (lower panels). The scale bar indicates 2 μm. **c** Average SypHy responses in control cells (scramble-shRNA, black) and in cells transfected with two independent shRNAs for Stx7 (Stx7-shRNA#1 or Stx7-shRNA#2 shown in red and blue, respectively). Note that SypHy responses were largely reduced by Stx7-shRNAs both at 10 Hz and 40 Hz, indicating an indispensable role of Stx7 in SV formation and maturation during development.

References for supplementary figures

1. Takamori, S. *et al.* Molecular anatomy of a trafficking organelle. *Cell* **127**, 831-846 (2006).
2. Wilhelm, B. G. *et al.* Composition of isolated synaptic boutons reveals the amounts of vesicle trafficking proteins. *Science* **344**, 1023-1028 (2014).
