# Supplementary material for: The endosomal Q-SNARE, Syntaxin 7, defines a rapidly replenishing synaptic vesicle recycling pool in hippocampal neurons
Source: Commun Biol. 2021 Aug 18;4:981. doi: 10.1038/s42003-021-02512-4 (PMC8373932; doi:10.1038/s42003-021-02512-4)
Supplement: Supplementary file 3 — Description of Supplementary Files [file 42003_2021_2512_MOESM3_ESM.pdf]

Description of Additional Supplementary Files

File name: Supplementary Data 1

Description: Detailed data for Figures 1e-h, 2c, d, 3d-f, 4, 5, 6c-g, and 7b, c.
