# Supplementary material for: The endosomal Q-SNARE, Syntaxin 7, defines a rapidly replenishing synaptic vesicle recycling pool in hippocampal neurons
Source: Commun Biol. 2021 Aug 18;4:981. doi: 10.1038/s42003-021-02512-4 (PMC8373932; doi:10.1038/s42003-021-02512-4)
Supplement: Supplementary file 5 — Reporting Summary [file 42003_2021_2512_MOESM5_ESM.pdf]

nature research

Corresponding author(s): Yasunori Mori & Shigeo Takamori

Last updated by author(s): Apr 5, 2021

Reporting Summary

Nature Research wishes to improve the reproducibility of the work that we publish. This form provides structure for consistency and transparency in reporting. For further information on Nature Research policies, see our Editorial Policies and the Editorial Policy Checklist.

Statistics

For all statistical analyses, confirm that the following items are present in the figure legend, table legend, main text, or Methods section.

n/a	Confirmed	
☐	☒	The exact sample size (n) for each experimental group/condition, given as a discrete number and unit of measurement
☐	☒	A statement on whether measurements were taken from distinct samples or whether the same sample was measured repeatedly
☐	☒	The statistical test(s) used AND whether they are one- or two-sided<br>*Only common tests should be described solely by name; describe more complex techniques in the Methods section.*
☒	☐	A description of all covariates tested
☒	☐	A description of any assumptions or corrections, such as tests of normality and adjustment for multiple comparisons
☐	☒	A full description of the statistical parameters including central tendency (e.g. means) or other basic estimates (e.g. regression coefficient) AND variation (e.g. standard deviation) or associated estimates of uncertainty (e.g. confidence intervals)
☐	☒	For null hypothesis testing, the test statistic (e.g. F, t, r) with confidence intervals, effect sizes, degrees of freedom and P value noted<br>*Give P values as exact values whenever suitable.*
☒	☐	For Bayesian analysis, information on the choice of priors and Markov chain Monte Carlo settings
☒	☐	For hierarchical and complex designs, identification of the appropriate level for tests and full reporting of outcomes
☒	☐	Estimates of effect sizes (e.g. Cohen's d, Pearson's r), indicating how they were calculated

Our web collection on statistics for biologists contains articles on many of the points above.

Software and code

Policy information about availability of computer code

Data collection	Live fluorescence images were collected with MetaMorph software (Molecular Devices). Fluorescence images obtained with a laser scanning confocal microscope were collected with ZEN software (Zeiss) or Fiji software. Western blot images were collected with Molecular Imager ChemiDoc (BioRad) . Immunoelectron micrographs were were taken with a CCD camera (Quemessa; Olympus-SIS).
Data analysis	Acquired live fluorescence images were analyzed using MetaMorph software (Molecular Devices). The rise phases were fitted with single exponential and time constants of the rise time were deduced by using the Solver function in Excel software. Band intensities in acquired images were quantified using Quantity One software (BioRad).

For manuscripts utilizing custom algorithms or software that are central to the research but not yet described in published literature, software must be made available to editors and reviewers. We strongly encourage code deposition in a community repository (e.g. GitHub). See the Nature Research guidelines for submitting code & software for further information.

Data

Policy information about availability of data

All manuscripts must include a data availability statement. This statement should provide the following information, where applicable:

- Accession codes, unique identifiers, or web links for publicly available datasets
- A list of figures that have associated raw data
- A description of any restrictions on data availability

The experimentally data that support the findings of this study are available from the corresponding authors upon reasonable request.

Field-specific reporting

Please select the one below that is the best fit for your research. If you are not sure, read the appropriate sections before making your selection.

- [x] Life sciences
- [ ] Behavioural & social sciences
- [ ] Ecological, evolutionary & environmental sciences

For a reference copy of the document with all sections, see nature.com/documents/nr-reporting-summary-flat.pdf

Life sciences study design

All studies must disclose on these points even when the disclosure is negative.

Sample size	No statistical methods were used to predetermine sample size. However, sample sizes are consistent with those reported in previous publications from our laboratory (Egashira et al., J. Neurosci. 2015, Egashira et al., PNAS 2016) and equivalent studies from other laboratories using pHluorin as a probe.
Data exclusions	No data were excluded from this study
Replication	All experimental findings were reliably reproduced.
Randomization	Randomization was not performed in this study.
Blinding	Blinding was not used in this study.

Reporting for specific materials, systems and methods

We require information from authors about some types of materials, experimental systems and methods used in many studies. Here, indicate whether each material, system or method listed is relevant to your study. If you are not sure if a list item applies to your research, read the appropriate section before selecting a response.

Materials & experimental systems

n/a	Involved in the study	
[ ]	[x]	Antibodies
[ ]	[x]	Eukaryotic cell lines
[x]	[ ]	Palaeontology and archaeology
[ ]	[x]	Animals and other organisms
[x]	[ ]	Human research participants
[x]	[ ]	Clinical data
[x]	[ ]	Dual use research of concern

Methods

n/a	Involved in the study	
[x]	[ ]	ChIP-seq
[x]	[ ]	Flow cytometry
[x]	[ ]	MRI-based neuroimaging

Antibodies

Antibodies used

Primary Antibodies
Anti-Syntaxin-7 (Bethyl Laboratories, A304-512A, Bethyl Laboratories)
Anti-GFP (Rabbit polyclonal, kindly gifted from Reinhard Jahn)
Anti-Synaptobrevin 2 (Mouse monoclonal, Cl69.1, kindly gifted from Reinhard Jahn)
Anti-Synaptophysin (Mouse monoclonal, Cl7.2, kind gifted from Reinhard Jahn)
Anti-Synaptophysin (Guinea pig polyclonal, 101 004, Synaptic Systems)
Anti-MAP2 (Chick polyclonal, ab5392, Abcam)
Anti-Tubulin beta3 (Mouse monoclonal, clone Tuj1, #MMS-435P, Covance)
Anti-Bassoon (Mouse monoclonal, SAP7F407, Novus Biologicals)

Secondary antibodies
Alexa-488-conjugated anti-rabbit IgG (A27034, Invitrogen)
Alexa-555-conjugated anti-mouse IgG (A28180, Invitrogen)
Alexa-633-conjugated anti-chick IgG (A11039, Invitrogen)
Alexa-633-conjugated anti-guinea pig IgG (A21005, Invitrogen)
HRP-linked anti-mouse IgG (kindly gifted from Reinhard Jahn)
HRP-linked anti-rabbit IgG (NA934-1ML, GE Healthcare)
FluoroNanogold-anti-rabbit Fab'-Alexa Fluo 488 (7204, Nanoprobes)

Validation

The specificity of the Stx7 antibody was confirmed in independent experiments where Stx7 expression was reduced by specific shRNAs (supplementary figure. 13).

Anti-GFP (https://www.sysy.com/products/gfp/facts-132002.php)
Anti-Synaptobrevin 2 (https://www.sysy.com/products/s-brevin2/facts-104211.php)
Anti-Synaptophysin (cl7.2) (https://sysy.com/product/101011)
Anti-Synaptophysin (101 004) (https://sysy.com/product/101004)
Anti-MAP2 (https://www.abcam.co.jp/map2-antibody-ab5392.html)
Anti-Tubulin beta3 (https://www.biolegend.com/en-us/products/purified-anti-tubulin-beta-3-tubb3-antibody-11580)
Anti-Bassoon (https://www.enzolifesciences.com/ADI-VAM-PS003/bassoon-monoclonal-antibody-sap7f407/)

Eukaryotic cell lines

Policy information about cell lines

Cell line source(s)	HEK-293T cells
Authentication	none
Mycoplasma contamination	none
Commonly misidentified lines (See ICLAC register)	none

Animals and other organisms

Policy information about studies involving animals; ARRIVE guidelines recommended for reporting animal research

Laboratory animals	Primary hippocampal cultures were prepared from embryonic day 16 ICR mice.
Wild animals	This study did not involve wild animals.
Field-collected samples	This study did not involve samples collected from the field.
Ethics oversight	Animals were treated according to our institutional guidelines for the care and use of animals (Doshisha University).

Note that full information on the approval of the study protocol must also be provided in the manuscript.
